# Revisiting the free radical theory using next-generation sequencing technology

**DOI:** 10.18632/aging.100188

**Published:** 2010-08-16

**Authors:** William C. Burhans, Martin Weinberger

**Affiliations:** Departmentof Molecular and Cellular Biology, Roswell Park Cancer Institute, Buffalo NY 14263, USA

The role of oxidative stress in aging proposed by the free radical theory has been the focus of investigations for more than fifty years.  The results of a large number of these investigations provide support for this theory.  However, numerous recent findings point to an unexpected complexity in the relationships between oxidative stress and aging.  This complexity is highlighted by the discovery by Timmermann et al. described in this issue of *Aging* [1] that a mutation in Tsa1p, a key element of oxidative stress defenses in the model organism budding yeast, shortens lifespan in concert with enhanced resistance to oxidative stress.  In addition to the implications of this finding for understanding aging, identification of this mutation by massively parallel sequencing of whole genomes emphasizes the enormous utility of next-generation sequencing technologies as investigative tools that will likely revolutionize genetics.

The starting point for the Timmermann et al. study was random mutagenesis of yeast cells that were subsequently screened for resistance to cumene hydroperoxide (CHP), an oxidizing agent employed in industrial processes and in studies of oxidative stress responses in yeast and other organisms.  The authors isolated a mutant strain resistant to CHP and another oxidizing agent, (tert-butyl hydroperoxide). Classical genetics approaches determined that this phenotype was inherited as a dominant allele in a monogenic fashion independently of genetic background.

Even "the awesome power of yeast genetics" is not sufficiently powerful to tackle some experimental challenges. For a variety of reasons, dominant mutations conferring resistance to stresses are difficult to identify using classic yeast genetics approaches, including those that employ strategies based on complementation using clone libraries.  Timmermann et al. approached this task instead by sequencing the genomes of the mutant strain and its wild type parent using the Roche 454 massively parallel sequencing platform.  Whole-genome sequencing using next-generation sequence technology was also recently employed to identify mutations underlying Mendelian diseases in humans [2]. The Timmermann et al. study is the first to apply this technology to map mutations in randomly mutagenized yeast, including strains with phenotypes that are not easily identified by classic functional genetics approaches.  Subtraction of the surprisingly large number of sequence variants found in both the wild type and mutant genomes, but not in a previously sequenced reference genome (as well as identification of non-uniform sequences and alignment artifacts) allowed Timmermann et al. to quickly narrow their search to four candidate mutations predicted to cause amino acid changes in proteins. Phenotypic analysis of cloned sequences containing each of these mutations revealed that *TSA1-B7*, a dominant allele of *TSA1* encoding a peroxiredoxin, was responsible for increased resistance to CHP.  Tsa1p catalyzes H_2_O_2_ reduction and acts as a molecular chaperone, and disruption of Tsa1p shortens replicative lifespan (RLS) and confers sensitivity to a variety of oxidants, including hydrogen peroxide and CHP [3]. The increased resistance to CHP conferred by the *B7* allele thus establishes it as a gain-of-function mutation.

Although resistance to oxidative stress is often associated with lifespan extension (as predicted by the free radical theory), surprisingly, strains harboring the *B7* mutation shortened, rather than lengthened RLS.  This is reminiscent of the findings of several recent studies that were similarly discordant in the connections they established between oxidative stress and predictions of the free radical theory.  For example, under glucose-restricted conditions *C. elegans *lifespan correlates with increased oxidative stress [4]. Although catalase inactivation shortens RLS of budding yeast [5], it lengthens the chronological lifespan of this organism in a different model of yeast aging in concert with elevated levels of hydrogen peroxide and increased oxidative damage. Similar enhancement of chronological lifespan by hydrogen peroxide is detected in calorie-restricted cells [6]. Perhaps most dramatic is the approximately 10-fold increase in the lifespan of naked mole rats compared to mice accompanied by high levels of oxidative damage [7].

In some cases, the apparent disconnect between experimental results and predictions of the free radical theory is related to hormesis effects that elevate oxidative and other stress defenses in response to low levels of oxidative stress [8]. Interestingly, although the *B7* mutation in *TSA1* confers resistance to CHP, Timmermann et al. also show that cells harboring this mutation are sensitive to hydrogen peroxide.  This finding is more in line with the shorter RLS of these cells and with the free radical theory.  Perhaps hydrogen peroxide accumulates in the B7 mutant and shortens RLS, similar to the effects on RLS of inactivating catalases, but triggers stress responses that protect against CHP. The more transparently clear lesson here is that not all forms of oxidative stress are equivalent in their effects on aging.  This isn't surprising in the context of the multitude of pathways that respond to different forms of oxidative stress [9] and the numerous mechanisms by which oxidants can modify different macromolecular targets.  Whatever the explanation, the findings by Timmermann et al. emphasize the enormous complexity of relationships between oxidative stress and aging. They also illustrate the awesome potential of next-generation sequencing technology combined with classical yeast genetics to make sense of the complexity revealed by these and other findings.
